# Medical Society of the County of Albany, Semi-Annual Meetiug, June 11, 1872

**Published:** 1872-07

**Authors:** James S. Bailey


ART. III—*Medical Society of the County of Albany. Semi-Annual Meeting, June 11th 1872. Reported by* JAMES S. BAILEY, M. D.

Dr. JOSEPH LEWI, President, in the Chair.

Dr. JAMES P. BOYD, Chairman of the Board of Censors, presented the names of the following gentlemen for admission to the Society.

Drs. F. C. Curtis, Isaac De Zouch, Wm. Hailes, S. A. Ingham, C. Lyon, J. H. Lasher, P. J. McGuire, B. U. Steenburgh, J. B. Stonehouse, Jr., Willis G. Tucker, E. Van Slyke, Wm. J. Warren.

They were admitted members on complying with the By-Laws.

Dr. CRAIG, moved that the names of Drs. Wand, Fisk and Starkweather, be referred back to the Board of Censors, inasmuch as they had not presented their credentials to the chairman —carried.

The Vice President, Dr. AMOS FOWLER, then delivered a short address, in which adverting to the duties of the Vice President, he promised at some future meeting of the society, to address them on the subject of Cerebro-Spinal Meningitis.

Dr. J. V. P. QUACKENBUSH, offered the following:

*Resolved,* That the thanks of this Society be presented to Dr Fowler for his recognition of the duty of the Vice President, to deliver a semi-annual address, and we hope that his promise to give us a paper on Cerebro-Spinal Meningitis, will be fulfilled on an early day. Adopted.

The Secretary, Dr. JOHN M. BIGELOW, then read a communication from a member of the society, Dr. J. V. R. Lansingh, Jr., of

Hartford, Indiana. It was entitled "An interesting case of Urethro-Vaginal Fistula, resulting from prolonged Labor." The report read as follows:

The following case is one of exceeding interest as well for the the rarity of its occurrence as the results which have rewarded a patient faith in nature's ability to cure and restore. To a young obstetrician, rupture of the perineum and fistulæ, offer severe discouragements which are scarcely lessened by wealth of counsel and the assistance of men of large experience, but in the unsettled West, where the principles of practice are decidedly crude, and the profession so much in dispute that it is a synonym for mere pretensions and lack of ability, it becomes a matter of grave responsibility and anxiety. Mrs. G., a healthy, well-formed brunette of 20, and a primipara was taken in labor on the 5th day of April, 1872, at 6 P. M. My partner was first called in, and remained in constant attendance. At 11 P. M. of that day, the membranes were ruptured, the os having dilated very slowly under continued and vigorous pains. The parts were soft, moist and seemingly favorable, and the head rapidly descended to the inferior strait, the occiput in the left acetabulum, and extension commenced, but was soon arrested. The perineum was rigid and unyielding, and the coccyx also which effectually resisted every attempt to produce extension with the finger passed in the rectum. Strong uterine contractions now came on, which wedged the head firmly in the pelvic walls, the vertex protruding about an inch. In this position it remained. All efforts to relax the rigid perineum failed and the uterine contractions gradually grew less frequent until they ceased. An inhalation of chloroform was given to relax the parts, but without effect. The usual means for producing contraction of the uterus were resorted to, in hopes of gaining ground by steadily forcing the child downward, but these all failed, and at 8 o'clock P. M. on the 6th inst., I was called in to try the vectis, (there being no forceps in the town,) and if failing in that, to perform craniotomy. An examination disclosed the condition I have stated. I could not introduce the vectis with facility, so ordered chloroform again administered, and in five minutes there was enough relaxation to allow a finger being introduced, which passing in the axilla, permitted sufficient traction to bring away the child. It was a large, well developed child, weighing some ten pounds, but had evidently been dead some hours. The placenta came away almost immediately, and the uterus contracted hard and firm under the hand pressed on the abdomen. The bladder was greatly distended, but although the parts were swollen and much tumefied, I readily passed the catheter and drew off a quart of urine. The patient rested well after her labor and manifested no untoward symptoms, until the second day, when she was attacked with puerperal fever, which however readily gave way to appropriate treatment. On the third day the desire to pass water ceased, which fact was but incidentally noticed by the attending physician, for this reason; the patient had been for some years suffering with severe hemorrhoids which caused frequent desire to go to stool, and she supposed that urine passed naturally at those times though there was no indication in the form of desire, so, when questioned as to that condition, she said it was all right and only once mentioned that there was a lack of desire. After some two weeks had passed, everything going well apparently during that time, she mentioned the fact of there being great heat and pain in the vagina, and said that the bladder appeared to be distended. I at once attempted to pass the cartheter and not only failed in that but was equally unsuccessful in an endeavor to find either the meatus or the urethra which generally is easily felt through the walls of the vagina running up behind the pubic bones. More than this I found the vagina filled with a mass of soft flabby membrane, which at first I supposed indicated a condition of recto-cele, but this opinion was found to be erroneous, on passing my finger in the rectum. I immediately explained to the patient that there was an unusual condition of affairs, which from my inability to diagnose, would necessitate an exposure. This was acceded to and with my partner I commenced a direct examination of the parts. The mass of membrane before mentioned, protruded from the labia and bore a black appearance, indicating a gangenous condition. This came away on slight traction and showed that sloughing was going on somewhere. Prosecuting our search further, we found an absence of a large part of the anterior wall of the vagina, the meatus urinarrius and nearly all of the urethra. Everything was congested, swollen and so much transformed, that at that time it was impossible to estimate the amount of loss to the substance there might be. Here then was a fistula, large in extent and annoying in the probability of permanent continuance. There was also loss of power in the sphincter vesicæ, which permitted a constant escape of urine into the vagina. Knowing the location of the fistula, of course a catheter could now be passed, but it was deemed impossible to retain it there, so for the time that idea was abandoned. A supporting treatment was at once instituted and the sheet anchor of dependence agreed upon in the free use of carbolic acid, gr v to the ℥. Shortly after, some obstinate symptoms led to a more complete examination, which resulted in finding a retroflexion of the uterus. This was restored by the sound and has not since caused trouble. Inflammmation subsided, but slowly, and nearly a month passed before the parts became normal in size and relations. An extended observation was then made, with the assistance of an impromptu parody on Sims speculum, and revealed the following conditions: the vagina normal except at the point of the fistula, which was over an inch long by a half inch in width, average estimate. All signs of the meatus and most of the urethra had disappeared, except a shallow sulcus or groove which marked the spot they had occupied. From the bladder a half inch, or perhaps less of the urethra projected forwards, distended sufficiently to allow my forefinger to be passed in the bladder. The fistulous opening was granulating well, and gave good promise of a complete restoration of the vaginal wall. For the urethra that much cannot be hoped. The sphincter muscle of the bladder still retains its paralyzed condition in spite of continued treatment. Can the prognosis of that condition be at all favorable? For two weeks the patient has worn a gum elastic catheter fastened in position, and through this the urine slowly passes as its secretes, a source of perpetual annoyance, a discouragement which produces unfavorable despondency. During all this time the general health has been built up and she sleeps well, eats very heartily, and though she sits up and walks around the room, yet she is disinclined to exercise. I can find no account of a similar case, though urethro-vaginal fistulæ are far from being rare, yet it is seldom that destruction of tissue proceeds so far that the entire urethra disappears. The cause of this seems evident in the long continued pressure of the child's head, but Thomas speaks of puerperal vaginitis as frequently being a cause. I think pressure was the cause in this case, 1st. because the urine passed involuntarily as early as the third day, and secondly, because sloughing went on very slowly with no marked symptoms of vaginitis inside of two weeks after accouchment, but the the puerperal condition may have hastened the work. Henceforth the urine will pass directly into the vagina, and be discharged from there in irregular and uncertain directions, excoriating the limbs and necessitating a relapse into the most important garment of infancy. Will it seriously disturb the vaginal or uterine functions, or do I fail to see advantages which might arise from intelligent exercise of operative interference, are questions which I leave to the discretion, sapientia et experientia of the gentlemen of the society.

*Hartford City, Indiana, June 4th,* 187[illegible].

Dr. JAMES S. BAILEY, moved that the Secretary be directed to thank Dr. Lansingh, for his communication. Carried.

The following names were proposed for membership, Drs. James P. Boyd, Jr., John A. LaGrange and Gilbert Ullman.

The Society then adjourned.

